# Cystatin D (CST5): An ultra-early inflammatory biomarker of traumatic brain injury

**DOI:** 10.1038/s41598-017-04722-5

**Published:** 2017-07-10

**Authors:** Lisa J. Hill, Valentina Di Pietro, Jon Hazeldine, David Davies, Emma Toman, Ann Logan, Antonio Belli

**Affiliations:** 1 Neuroscience & Ophthalmology Research Group, Institute of Inflammation & Ageing, College of Medical and Dental Sciences, University of Birmingham, Edgbaston, B15 2TT Birmingham UK; 2 National Institute for Health Research Surgical Reconstruction and Microbiology Research Centre, Queen Elizabeth Hospital, Edgbaston, B15 2TH Birmingham UK

## Abstract

Traumatic brain injury (TBI) is set to become the leading cause of neurological disability across all age groups. Currently, no reliable biomarkers exist to help diagnose the severity of TBI to identify patients who are at risk of developing secondary injuries. Thus, the discovery of reliable biomarkers for the management of TBI would improve clinical interventions. Inflammatory markers are particularly suited for biomarker discovery as TBI leads to very early alterations in inflammatory proteins. Using the Proseek Multiplex Inflammation assay, we measured in patients that had suffered mild TBI (n = 10) or severe TBI (n = 10) with extra-cranial injury or extracranial injury only (EC) (n = 10), 92 inflammation-associated proteins in serum obtained: <1 hr (within 1-hour), 4–12 hr and 48–72 hr post injury. Changes were compared to healthy volunteers (HV). Our results identified CST5, AXIN1 and TRAIL as novel early biomarkers of TBI. CST5 identified patients with severe TBI from all other cohorts and importantly was able to do so within the first hour of injury. AXIN1 and TRAIL were able to discriminate between TBI and HV at <1 hr. We conclude that CST5, AXIN1 and TRAIL are worthy of further study in the context of a pre-hospital or pitch-side test to detect brain injury.

## Introduction

Traumatic brain injury (TBI) is the leading cause of death and disability among young adults. In the US, TBI has been called “the silent epidemic”^1^, where relatively young victims (mean, 29.5 years of age) pose a tremendous burden to families and society in terms of years of lost productivity and increased demands on the healthcare system^2^. According to the World Health Organization, by 2020 TBI will become the world’s leading cause of neurological disability across all age groups.

The pathology of TBI, described as a primary injury, results from mechanical damage to neural and vascular structures together with a progressive cascade of molecular secondary events leading to secondary injuries^3^ that impair function, damage other brain structures and promote further cell death^4,5^.

Whilst improvements in emergency response times have increased TBI survivability, the necessity for discovering reliable markers by which to identify patients at risk of the development of secondary injuries and thus requiring more active monitoring and intervention remains a significant challenge.

Currently, few reliable indicators of prognosis exist for use in TBI, the Glasgow Coma Scale (GCS), pupil reactivity, and head computed tomography (CT)^6^. These indicators have proven useful for patient stratification but have limited use for predicting the development of secondary injury events. This is particularly true for patients with mild TBI (mTBI) which represents approximately 80% of all TBI cases and, where post-concussive symptoms including headaches, sleep disturbances, nausea, impaired attention, and memory problems may develop but not be mentioned due to limited patient follow up^7–9^. Thus, there is an urgent need to identify biomarkers that will assist in patient diagnosis and prognosis that correlate with the biological response to the injury, which can be easily measured in a non-invasive manner from samples such as peripheral blood and finally, can be detected early after TBI in order to predict probability of onset of secondary damage. To date, the majority of TBI biomarker research has focused on protein profiling with the BB isozyme of creatine kinase (CK-BB)^10^, glial fibrilary acidic protein (GFAP)^11^, myelin basic protein (MBP)^12^, neuron-specific enolase (NSE)^13^, and S100B^14^ being the most studied as potential candidate biomarkers of TBI, albeit of limited use for discriminating those patients vulnerable to secondary adverse outcomes. Accordingly, many studies have failed to show reproducible results for these candidate biomarkers of TBI, particularly in the case of S100B during polytrauma, as this protein is not brain specific and may derive from several extracranial sources^15,16^. There are however, some more promising axonal injury biomarkers, including Tau^17^ and Neurofilament light protein^18^ which have performed better than other candidate biomarkers mentioned previously by being able to correlate with clinical findings and predict clinical outcomes.

It has been demonstrated that within minutes of a traumatic impact a robust inflammatory response is elicited in the injured brain^19^. Neuroinflammation is responsible for both beneficial and detrimental effects, contributing to secondary brain damage but also facilitating neurorepair. For these reasons, these inflammatory molecules could satisfy the three main characteristics required for a biomarker of the diagnosis and prognosis of TBI. In particular, identification of these early phase severity-related inflammatory biomarkers could permit clinicians to recognise and treat those patients at risk of secondary neural damage while they are still capable of responding to therapy before irreversible damage occurs. In addition, being able to stratify patients for injury severity and those who are at risk from secondary damage would also avoid the use of unnecessary medical or surgical interventions in all patients with TBI.

In this novel study we have identified biomarkers of TBI at different time points, including samples which were collected within 1 hour from injury by the pre-hospital clinical team at the scene of injury prior to transfer to a regional major trauma centre (MTC). Using a panel of 92 inflammation-associated human proteins which were screened simultaneously using the Proseek Multiplex Inflammation I assay. The serum biomarkers were analysed from patients with mild TBI (mTBI) with extracranial injury (EC), severe TBI (sTBI) with EC and EC only and all groups were compared to control patients (healthy volunteers; HV).

## Results

### Temporal protein expression within each of the injury groups and compared to HVs

**Figure 1:**
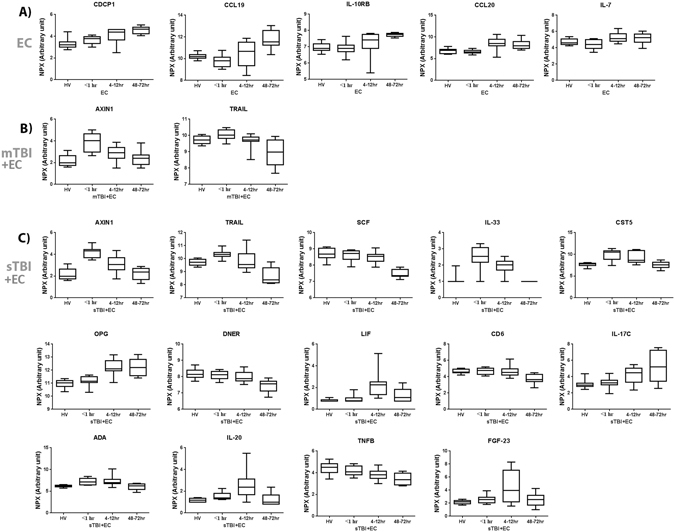
Comparison of temporal protein expression within each of the injury groups. Within injury groups of EC patients, mTBI+EC patients and sTBI+EC patients, expression of inflammatory proteins significantly differed between HV, <1 hr, 4–12 hr and 48–72 hr time points.

For the EC injury group only, 28 of the 92 inflammatory proteins measured were significantly separated by ANOVA across time (Table 1A). Of these 28 proteins, 5 proteins were temporally separated only within the EC group and did not demonstrate a time course difference in proteins levels from the serum of HV or TBI patients. These five proteins (Fig. 1A) were CUB domain containing protein 1 (CDCP1) (p < 0.0001), C-C motif chemokine ligand 19 (CCL19) (p < 0.0001), Interleukin 10 receptor beta (IL-10RB) (p < 0.0001), C-C motif chemokine ligand 20 (CCL20) (p < 0.0001) and Interleukin 7 (IL-7) (p < 0.001). ANOVA yielded 14 and 37 significantly separated proteins between time points and when compared to HV in the mTBI+EC (Table 1B) and sTBI+EC (Table 1C) groups respectively, but 12 proteins in the mTBI+EC and 23 in the sTBI+EC groups were also detected to be temporally different in the EC groups and therefore were not suitable to detect early inflammatory biomarkers for severity of TBI.

**Table 1:** Inflammatory proteins differentially expressed over time for each injury group.

Protein	P-Value
**A**) **EC**	
IL-8	<0.0001
IL-10	<0.0001
SIRT2	<0.0001
MCP-3	<0.0001
IL-6	<0.0001
OSM	<0.0001
HGF	<0.0001
TGFA	<0.0001
STAMPB	<0.0001
CASP-8	<0.0001
**CDCP1**	<**0.0001**
EN-RAGE	<0.0001
**CCL19**	**<0.0001**
TRANCE	<0.0001
CSF-1	<0.0001
IL-18R1	<0.0001
4E-BP1	<0.0001
**IL-10RB**	<**0.0001**
CCL23	<0.0001
CX3CL1	<0.0001
**CCL20**	<**0.0001**
MCP-1	<0.0001
**IL-7**	<**0.0001**
TWEAK	<0.0001
MMP-10	<0.0001
ST1A1	<0.0001
CD244	<0.0001
FGF-21	<0.0001
**B) mTBI+EC**	
ST1A1	<0.0001
CASP-8	<0.0001
SIRT2	<0.0001
STAMPB	<0.0001
IL-6	<0.0001
OSM	<0.0001
MCP-3	<0.0001
IL-10	<0.0001
TRANCE	<0.0001
4E-BP1	<0.0001
HGF	<0.0001
TGFA	<0.0001
**AXIN1**	<**0.0001**
**TRAIL**	<**0.0001**
**C) sTBI+EC**	
TWEAK	<0.0001
4E-BP1	<0.0001
TRANCE	<0.0001
ST1A1	<0.0001
IL-8	<0.0001
MCP-3	<0.0001
IL-6	<0.0001
**AXIN1**	<**0.0001**
OSM	<0.0001
**SCF**	<**0.0001**
HGF	<0.0001
SIRT2	<0.0001
CASP-8	<0.0001
STAMPB	<0.0001
**TRAIL**	<**0.0001**
IL-10	<0.0001
**IL-33**	<**0.0001**
**CST5**	<**0.0001**
IL-18R1	<0.0001
MCP-1	<0.0001
EN-RAGE	<0.0001
**OPG**	<**0.0001**
**DNER**	<**0.0001**
TGFA	<0.0001
MMP-10	<0.0001
**LIF**	<**0.0001**
**CD6**	<**0.0001**
FGF-21	<0.0001
**IL-17C**	<**0.0001**
**ADA**	<**0.0001**
CD244	<0.0001
CX3CL1	<0.0001
CCL23	<0.0001
**IL-20**	<**0.0001**
**TNFB**	<**0.0001**
CSF-1	<0.0001
**FGF-23**	<**0.0001**

Proteins exclusively found to differ between time points are shown for each group; EC (**in bold**), mTBI + EC (**in bold**) and sTBI + EC (**in bold**). ANOVA with Bonferroni correction to show differences within each group between time points (*p* < *0.00057*).

When looking at temporal changes in the each of the separate groups, AXIN1 (p < 0.0001) and tumour necrosis factor superfamily member 10 (TRAIL) (p < 0.001) (Fig. 1B and C) demonstrated peak levels at <1 hr and they were the only two proteins found to demonstrate temporal changes exclusively in the TBI groups, although neither of these proteins could discriminate between mTBI+EC and sTBI+EC (Table 1B and C). The temporal expression of AXIN1 and TRAIL was not altered in the EC injury group.

Exclusively in the sTBI+EC groups, 12 of the 37 protein’s levels were found to significantly differ over time, yielding a candidate cohort of biomarkers for sTBI (Table 1C). These 12 sTBI biomarkers (Fig. 1C) were Stem cell factor (SCF) (p < 0.0001), Interleukin-33 (IL-33) (p < 0.0001), Cystatin D (CST5) (p < 0.0001), Osteoprotegerin (OPG) (p < 0.0001), Delta and Notch-like epidermal growth factor-related receptor (DNER) (p < 0.0001), Leukemia inhibitory factor (LIF) (p < 0.0001), T cell surface glycoprotein CD6 isoform (CD6) (p < 0.0001), Interleukin-17C (IL-17C) (p < 0.001), Adenosine Deaminase (ADA) (p < 0.0001), Interleukin-20 (IL-20) (p < 0.001), TNF-beta (TNFB) (p < 0.001) and FGF-23 (p < 0.001).

### Proteins differentially expressed in the serum from the TBI and EC injury groups at each time point

**Figure 2:**
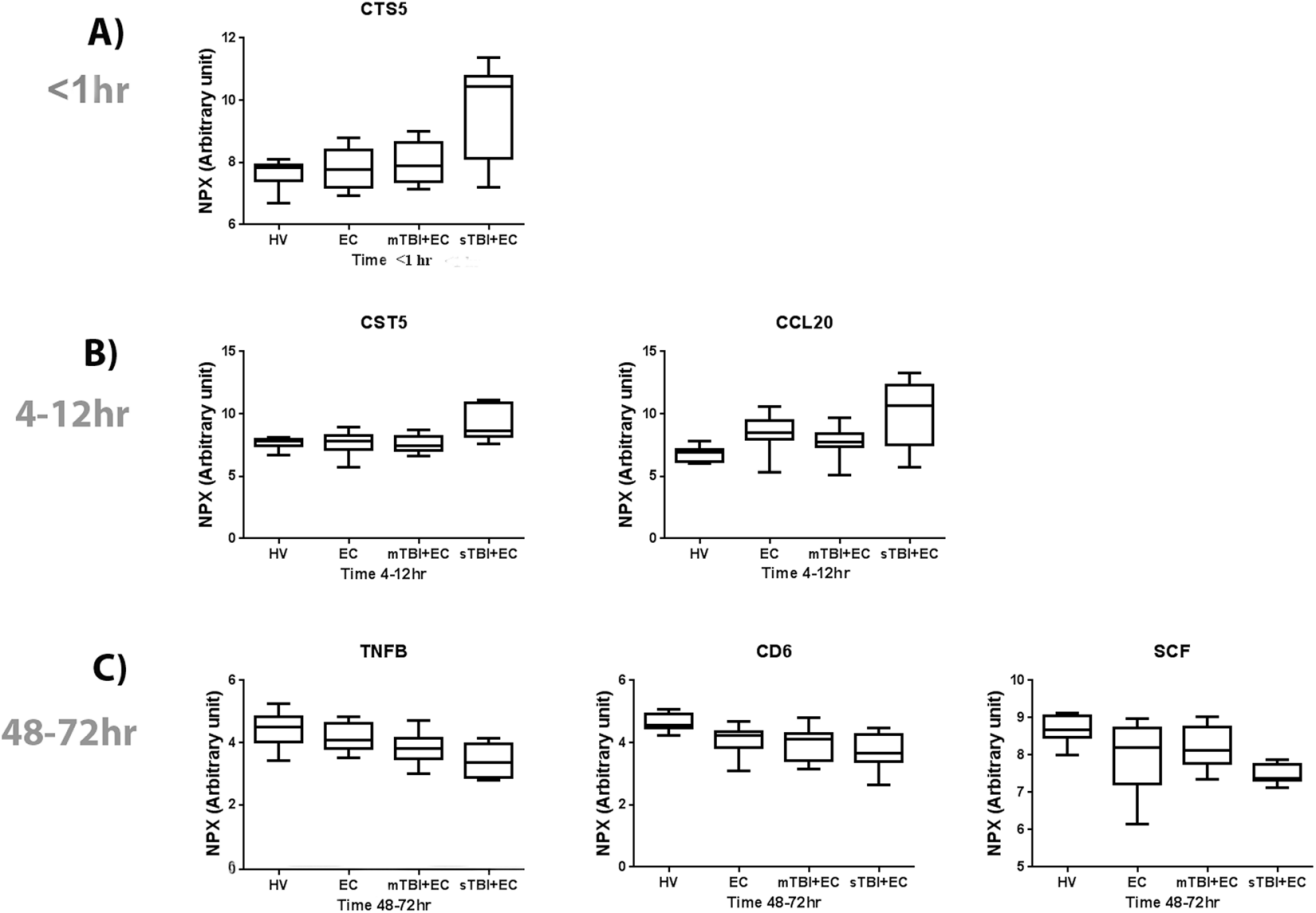
Comparison of protein expression between HV and injury groups at each time point. Candidate biomarkers are shown at <1 hr (**A**), 4–12 hr (**B**) and 48–72 hr (**C**).

At <1 hr, 12 proteins were differentially expressed between HV and injury groups (Table 2A). From these 12 proteins, CST5, is evidenced in Table 2C as a potential biomarker of sTBI+EC as serum levels were increased after sTBI (p < 0.0001) when compared to levels in patients with diagnosed mTBI+EC and, furthermore, was at its highest level within the <1 hr sample (Fig. 2A). CST5 levels were also raised (and still higher than mTBI, p < 0.002) at the 4–12 hr time point but was not different to HV in the 48–72 hr time point (Fig. 2B). Interestingly, from the 18 significantly different proteins detected within the 4–12 hr time point across all injury and HV groups (Table 2B), only CST5 and CCL20 were also present in the panel of proteins differentially expressed across all the time points (Table 2). In particular, CCL20 was exclusively found in serum from the 4–12 hr time point with increasing levels in the sTBI+EC group when compared to levels in the HV (p < 0.001) and mTBI+EC groups. However, this protein was not significantly different between the EC and the either of the TBI injured groups (Fig. 2B). At the 48–72 hr time point, 19 serum proteins were significantly different between HV and injury groups (Table 2C) and of these, TNFB (p < 0.0001), CD6 (p < 0.001) and SCF (p < 0.001) were correlated with the presence of TBI and also the severity of TBI (Fig. 2C). TNFB levels showed no differences between HV and EC groups (p = 0.44), between EC and sTBI+EC groups (p = 0.21) or between mTBI+EC and sTBI+EC groups (p = 0.639) but there were differences in serum levels between HV and mTBI+EC patients (p < 0.0001), HV and sTBI+EC patients (p < 0.01) and EC and mTBI+EC patients (p < 0.01) demonstrating a difference in expression between injury groups and HV but not between TBI groups (Fig. 2C). Serum CD6 levels were decrease after EC (p < 0.02), mTBI+EC (p < 0.01) and sTBI+EC (p < 0.001) compared to HV, however there were no differences between EC, mTBI+EC and sTBI+EC suggesting, as with TNFB, it was not an ideal biomarker for TBI (Fig. 2C). SCF levels were lower between HV and EC (p = 0.047) or sTBI+EC (p < 0.001) injury groups, but interestingly levels of SCF were not different between HV and mTBI+EC (p = 0.2866) injured patients (Fig. 2C).

**Table 2:** Inflammatory proteins which differed between extracranial, mTBI+EC and sTBI+EC across different time points.

Protein	P-Value
**A) <1hr**	
AXIN1	<0.0001
IL-6	<0.0001
SIRT2	<0.0001
CASP-8	<0.0001
ST1A1	<0.0001
STAMPB	<0.0001
**CST5**	**<0.0001**
IL-10	<0.0001
4E-BP1	<0.0001
IL-8	<0.0001
EN-RAGE	<0.0001
uPA	<0.0001
**B) 4–12 hr**	
IL-8	<0.0001
MCP-3	<0.0001
IL-6	<0.0001
OSM	<0.0001
HGF	<0.0001
IL-10	<0.0001
TGFA	<0.0001
STAMPB	<0.0001
CX3CL1	<0.0001
CASP-8	<0.0001
SIRT2	<0.0001
MCP-1	<0.0001
EN-RAGE	<0.0001
FGF-21	<0.0001
**CST5**	**<0.0001**
IL-18R1	<0.0001
TRANCE	<0.0001
**CCL20**	**<0.0001**
**C) 48–72 hr**	
IL-6	<0.0001
MCP-3	<0.0001
OSM	<0.0001
IL-10	<0.0001
TGFA	<0.0001
CCL23	<0.0001
HGF	<0.0001
EN-RAGE	<0.0001
TRANCE	<0.0001
CSF-1	<0.0001
IL-18R1	<0.0001
TWEAK	<0.0001
IL-8	<0.0001
TNFSF14	<0.0001
MMP-10	<0.0001
FGF-21	<0.0001
**TNFB**	**<0.0001**
**CD6**	**<0.0001**
**SCF**	**<0.0001**

The inflammatory proteins that differed between HV, EC, mTBI+EC and sTBI+EC at <1 hr (A), 4–12 hr (B) and 48–72hr (C) are listed. Candidate biomarker proteins are shown **in bold**. (*Significance* = *P* < 0.00057).

## Discussion

In this paper we have analysed the expression profile of a panel of 92 different inflammatory proteins at different time points, following different grades of brain injury with extra-cranial injury. Although the inflammatory reaction triggered within seconds to minutes after traumatic insult has been well described, a better understanding about the magnitude, timing and duration of expression of some of these inflammatory mediators might be able to provide not only information about the nature and extent of brain injury but also may reflect the complexity of the subsequent tissue response deriving from multiple insults.

Neuroinflammation in the acute stage of TBI mobilizes immune cells, astrocytes, chemokines and cytokines^19,20^ towards the site of injury to mount an anti-inflammatory response. However, an excess activation of these inflammatory molecules can contribute to a secondary cell death in TBI^17^. Modulating these inflammatory elements from proinflammatory to antiinflammatory has been considered a suitable future strategy for therapeutic purpose^21,22^.

Among the proteins analysed in this study, several TBI-related cytokines, have been previously well characterized in terms of their potential role in neuronal responses and their potential to act as biomarkers of the extent and nature of the injury, including TNFα, IL-10, IL-6, IL-8. To date, IL-6 has been considered as the most promising molecule, since a 100 fold change can be readily measured in serum following TBI^23^. Moreover, IL-6 levels have previously been correlated with changes in ICP^24^ and outcomes^25^, but not in polytrauma patients where extracranial injury demonstrates effect as a significant confounder. Our data for T < 1 hr samples showed that serum IL-6 levels were also significantly and differentially expressed in patients with extracranial injury. In particular, results showed that serum IL-6 was able to discriminate between mTBI + EC and sTBI + EC but not between patients with mTBI + EC and EC only (data not shown). Only between 4–12 hrs did IL-6 expression increase significantly in the sTBI+EC compared to the mTBI+EC and EC groups. By 48–72 hrs IL-6 levels were similar in all injury groups (data not shown). Thus, not representing an ideal ultra-early TBI biomarker.

In Tables 1 and 2, we present a panel of inflammatory proteins that were significantly expressed in the serum of patients across the time points for each injury group and between the injury groups at specific time points. Understanding the kinetics of these proteins and the potential to discriminate between the three groups of patients has the potential to identify biomarkers for TBI.

In particular, in the mTBI+EC group AXIN1 and TRAIL (Fig. 1B) demonstrated a spike in levels within the first hour on injury followed by a steady decline over time. In the EC group AXIN1 and TRAIL did not show any significant temporal changes. AXIN1 is a cytoplasmic protein and a negative regulator of the WNT signalling pathway whereby it can induce apoptosis alter WNT signalling control of inflammation and wound healing^26,27^. This may be particularly important in the cases of TBI where early spikes (i.e. in the pre-hospital setting) of AXIN1 could result in higher levels of neuronal cell death later on via its negative effects on WNT signalling^27^. TRAIL is a protein which can induce apoptosis via activation of Caspase-8^28^ but it also activates the NF-κB pathway, which can induce inflammatory processes^29^, alter cellular functions^30^ and enhance synaptic plasticity and memory formation^31^. Therefore, in the case of TBI, decreasing levels of TRAIL over time may result in neuronal deficits as a result of a reduction in NF-κB signalling.

In the sTBI+EC group a cohort consisting of AXIN1, TRAIL, SCF, IL-33, CST5, OPG, DNER, LIF, CD6, IL-17, ADA, IL-20 and TNFB, FGF-23 were all differentially expressed over time. Here, and similar to the mTBI+EC group, there were significant increases in AXIN1 and TRAIL within the first hour of injury (Fig. 1C) followed by a steady decline over time. However, their ability to discriminate between mTBI and sTBI were not evident in this study and, therefore, they may only prove useful as a pre-hospital biomarker for brain trauma in general.

Among the cohort of proteins differentially expressed in sTBI+EC, CST5 showed the best characteristics as very early biomarker of TBI. CST5 can easily differentiate the severity of TBI. However, CST5 did not discriminate between the three groups, mTBI + EC, EC and HV. Nevertheless, the early detection of CST5 in early serum samples, make this protein a strong candidate biomarker to determine TBI when used in conjunction with clinical observations. CST5 is an inhibitor of lysosomal and secreted cysteine proteases^32^. It was originally purified from saliva^33^ and inhibits proliferation, migration, and invasion of colon carcinoma cells indicating a tumor suppressor activity that is unrelated to the protease activity^34^. Transcriptomic analysis has also showed that cystatin D might alter gene expression, including that of genes encoding transcription factors such as *RUNX1*, *RUNX2*, and *MEF2C* in HCT116 cells^34^. Beside the relatively undefined role of CST5, the novelty of the results reported here focusses on its clear association with TBI. Except for AXIN1, TRAIL and CST5, all the candidate biomarkers in the sTBI group were not evidently different in the T < 1 hr time point, therefore were not suitable to detect TBI at an ultra-early time point.

Early and objective pre-hospital detection of TBI would support clinical decision making and the correct triage of major trauma, which, in countries such as the US and UK, involves delivering significant neurotrauma cases to a level-1 trauma centre with neurosurgical facilities, as opposed to local trauma units. Moreover, the correct diagnosis of TBI, which is one of hardest diagnosis to make in medicine, would allow clinicians to implement strategies to reduce secondary brain injury at early stage, e.g. by optimising blood and oxygen delivery to the brain and avoid manoeuvres that could potentially increase intracranial pressure. In addition, this has potential implications for drug development, as novel compounds could be given immediately after injury and potentially commenced at the roadside, if there was sufficient confidence in the diagnosis of TBI.

In conclusion, this paper has described CST5, TRAIL and AXIN1 as potential biomarkers of TBI. CST5 has demonstrated the ability to differentiate between severely brain injured patients and those with either mild or no brain injury within the first hour. AXIN1 and TRAIL were able to discriminate between TBI severities within the first hour. Not only we have highlighted CST5, TRAIL and AXIN1 as new TBI biomarkers worthy of further investigation but we have described how a panel of markers have behaved over a 72 hour period. Although the Proseek Multiplex Inflammation assay is not suitable for a point of care testing device, CST5 shows to be an ideal ultra-early biomarker with its ability to not only to determine the presence of TBI but discriminate between TBI severities, which may be a very useful aid for the pre-hospital team at the road side if a new portable device can be developed. In addition, TRAIL and AXIN1 with their ability to show the presence of TBI should also undergo further interrogation in the search for a reliable road-side and pitch-side investigations to diagnose TBI.

## Methods

### Patients and study approval

Study participants were recruited through the Surgical Reconstruction and Microbiology Research Centre (SRMRC) at Queen Elizabeth Hospital of Birmingham (UK) as part of the RECOS (Ethics Ref. 11-0429AP28) and the Golden Hour studies (Ethics Ref. 13/WA/0399). Written informed consent was received from participants or valid proxy (family or a professional not directly involved in the study) prior to inclusion in the study. The study was approved by the National Research Ethics Service (Research Ethics Committee reference 13/WA/0399, Integrated Research Application System ID 125988). Both RECOS and Golden Hour studies comply with the guidelines of the Declaration of Helsinki. Patients were categorised into HV, EC, mTBI + EC and sTBI + EC. EC injury patients had radiographically or clinically-confirmed injuries, no history or signs of head trauma, and no current clinically significant infection, individuals with a history of neurological or psychiatric disorders were excluded. Mild TBI with EC included those patients with confirmed head trauma and Glasgow Coma Scale (GCS) score ≥13. Severe TBI with EC included patients with GCS ≤8. All patients were gender and age matched to HVs. Patient demographics are shown in Table 3. Blood samples from 10 patients in each category were obtained at different time points post injury: T < 1 hr (within 1 hr), T4–12 hr, T48–72 hr. Blood samples were not collected between 12–48 hours to adhere to the study protocol as our aims were to collect and investigate temporal changes between very early until later time points following injury.

**Table 3:** Demographic characteristics of the study participants.

Study group	Number of samples	Age			Gender	Mechanism of injury^a^					GCS		
		Mean	±SD	(Range)	M/F	A	F	P	RTC	B	Median	±SD	(Range)
HV	10	35.6	14.5	(18–52)	8/2	NA					NA		
mTBI+EC	10	42.6	24.4	(18–82)	9/1	0	5	0	4	1	14	1.7	(13–15)
sTBI+EC	10	36.3	16.5	(21–65)	7/3	1	0	1	8	0	4	1.7	(3–8)
EC only	10	32.3	9.8	(19–79)	8/2	2	2	1	3	2	NA		

^a^Mechanism of injury: A, assault; F, fall; P, penetrating; RTC, road traffic accident; B, other blunt; NA: Not Applicable.

### Sample processing

Peripheral blood samples were taken within the first hour (<1 hr) study patients by pre-hospital clinicians from the West Midlands Medical Emergency Response Intervention Team (MERIT) following initial intravenous access at the scene of injury. Further peripheral blood samples were taken from the same patients during subsequent hospital admission between 4–12 hr and 48–72 hr following the trauma. Once collected, blood samples were left at room temperature for 30 minutes prior to centrifugation at 3000 rpm for 10 minutes at 4 °C. Serum aliquots were stored at −80 °C until analysis. All samples were processed within two hours of venepuncture.

### Proximity extension assay

The Proseek Multiplex Inflammation I (Olink Bioscience, Uppsala, Sweden) was used to perform the multiplex proximity assay according to the manufacturers protocol (Olink Bioscience, Uppsala, Sweden). Briefly, 1 µl of human serum together with 3 µl of mix containing antibodies labelled with corresponding DNA oligonucleotides was incubated over night at 8 °C. Following this, 96 µl of extension mix containing proximity extension assay enzymes and PCR reagents were added. Following a 5′ incubation plates were placed on the thermal cycler for 17 cycles of DNA amplification. The 96.96 Dynamic Array IFC (Fluidigm, CA, USA) was primed according to the manufacturer’s instructions. In a separate plate, 7.2 µl of detection mix and 2.8 µl of samples were mixed together and from this, 5 µl was loaded into the primed 96.96 Dynamic Array IFC. The specific primer pairs for the 92 inflammatory proteins (Table 4) were loaded into the 96.96 dynamic array and the protein expression programme activated in the Fluidigm Biomark reader as per Proseek instructions. Further details about detection limits, reproducibility and validations can be found at the Olink webpage (http://www.olink.com/products/proseek-multiplex/downloads/data-packages).

**Table 4:** Biomarkers analysed with the Proseek proximity extension assay.

Biomarker		
Adenosine Deaminase (ADA)	Glial cell line-derived neurotrophic factor (hGDNF)	Monocyte chemotactic protein 1 (MCP-1)
Artemin (ARTN)	Hepatocyte growth factor (HGF)	Monocyte chemotactic protein 2 (MCP-2)
Axin-1 (AXIN1)	Interferon gamma (IFN-gamma)	Monocyte chemotactic protein 3 (MCP-3)
Beta-nerve growth factor (Beta-NGF)	Interleukin-1α (IL-1α)	Monocyte chemotactic protein 4 (MCP-4)
Brain-derived neurotrophic factor (BDNF)	Interleukin-10 (IL-10)	Natural killer cell receptor 2B4 (CD244)
C-C motif chemokine 19 (CCL19)	Interleukin-10 receptor subunit alpha (IL-10RA)	Neurotrophin-3 (NT-3)
C-C motif chemokine 20 (CCL20)	Interleukin-10 receptor subunit beta (IL-10RB)	Neurturin (NRTN)
C-C motif chemokine 23 (CCL23)	Interleukin-12 subunit beta (IL-12B)	Oncostatin-M (OSM)
C-C motif chemokine 25 (CCL25)	Interleukin-13 (IL-13)	Osteoprotegerin (OPG)
C-C motif chemokine 28 (CCL28)	Interleukin-15 receptor subunit alpha (IL-15RA)	Programmed cell death 1 ligand 1 (PD-L1)
C-C motif chemokine 3 (CCL3)	Interleukin-17A (IL-17A)	Protein S100-A12 (EN-RAGE)
C-C motif chemokine 4 (CCL4)	Interleukin-17C (IL-17C)	Signaling lymphocytic activation molecule (SLAMF1)
C-X-C motif chemokine 1 (CXCL1)	Interleukin-18 (IL-18)	SIR2-like protein 2 (SIRT2)
C-X-C motif chemokine 10 (CXCL10)	Interleukin-18 receptor 1 (IL-18R1)	STAM-binding protein (STAMPB)
C-X-C motif chemokine 11 (CXCL11)	Interleukin-2 (IL-2)	Stem cell factor (SCF)
C-X-C motif chemokine 5 (CXCL5)	Interleukin-2 receptor subunit beta (IL-2RB)	Sulfotransferase 1A1 (ST1A1)
C-X-C motif chemokine 6 (CXCL6)	Interleukin-20 (IL-20)	T cell surface glycoprotein CD6 isoform (CD6)
C-X-C motif chemokine 9 (CXCL9)	Interleukin-20 receptor subunit alpha (IL-20RA)	T-cell surface glycoprotein CD5 (CD5)
CDL40 receptor (CD40)	Interleukin-22 receptor subunit alpha-1 (IL-22 RA1)	Thymic stromal lymphopoietin (TSLP)
Caspase 8 (CASP-8)	Interleukin-24 (IL-24)	TNF-beta (TNFB)
CUB domain-containing protein 1 (CDCP1)	Interleukin-33 (IL-33)	TNF-related activation-induced cytokine (TRANCE)
Cystatin D (CST5)	Interleukin-4 (IL-4)	TNF-related apoptosis-inducing ligand (TRAIL)
Delta and Notch-like epidermal growth factor-related receptor (DNER)	Interleukin-5 (IL-5)	Transforming growth factor alpha (TGF-alpha)
Eotaxin-1 (CCL11)	Interleukin-6 (IL-6)	Tumor necrosis factor (Ligand) superfamily, member 12 (TWEAK)
Eukaryotic translation initiation factor 4E-binding protein 1 (4E-BP1)	Interleukin-7 (IL-7)	Tumor necrosis factor (TNF)
Fibroblast growth factor 19 (FGF-19)	Interleukin-8 (IL-8)	Tumor necrosis factor ligand superfamily member 14 (TNFSF14)
Fibroblast growth factor 21 (FGF-21)	Latency-associated peptide transforming growth factor beta 1 (LAP TGF-beta-1)	Tumor necrosis factor receptor superfamily member 9 (TNFRSF9)
Fibroblast growth factor 23 (FGF-23)	Leukemia inhibitory factor (LIF)	Urokinase-type plasminogen activator (uPA)
Fibroblast growth factor 5 (FGF-5)	Leukemia inhibitory factor receptor (LIF-R)	Vascular endothelial growth factor A (VEGF-A)
Fms-related tyrosine kinase 3 ligand (Flt3L)	Macrophage colony-stimulating factor 1 (CSF-1)	
Fractalkine (CX3CL1)	Matrix metalloproteinase-1 (MMP-1)	
	Matrix metalloproteinase-10 (MMP-10)	

### Data Analysis

Data were transferred from the Biomark reader to Olink Wizard for GenEx software (Olink). Data were presented as normalized protein expression units (NPX) on a linear scale with high NPX corresponding to high protein concentration. ANOVA with Bonferroni correction was applied and the P-value for significance was adjusted to 0.00057. For comparisons within each group a Tuckey Kramer post hoc test was performed and for comparisons of each group across time a Dunnett post hoc test was performed.
