# Author Correction: Cystatin D (CST5): An ultra-early inflammatory biomarker of traumatic brain injury

**DOI:** 10.1038/s41598-018-22951-0

**Published:** 2018-03-12

**Authors:** Lisa J. Hill, Valentina Di Pietro, Jon Hazeldine, David Davies, Emma Toman, Ann Logan, Antonio Belli

**Affiliations:** 1 Neuroscience & Ophthalmology Research Group, Institute of Inflammation & Ageing, College of Medical and Dental Sciences, University of Birmingham, Edgbaston, B15 2TT Birmingham UK; 2 National Institute for Health Research Surgical Reconstruction and Microbiology Research Centre, Queen Elizabeth Hospital, Edgbaston, B15 2TH Birmingham UK

Correction to: *Scientific Reports* 10.1038/s41598-017-04722-5, published online 10 July 2017

The original version of this Article contained a typographical error in the spelling of the author Emma Toman, which was incorrectly given as Emma Tomman. This has now been corrected in the PDF and HTML versions of the Article.

